# Factors associated with age at first sexual initiation among youths in Gamo Gofa, South West Ethiopia: a cross sectional study

**DOI:** 10.1186/1471-2458-13-622

**Published:** 2013-07-02

**Authors:** Marelign Tilahun, Gistane Ayele

**Affiliations:** 1 Department of Public Health, College of Medicine and Health Sciences, Arba-Minch University, P.O. Box: 21, Arba-Minch, Ethiopia

## Abstract

**Background:**

Early sexual initiation increases the risk of HIV infection and other sexually transmitted diseases. This study aimed to determine age at first sexual initiation and associated factors among youths in south west Ethiopia.

**Methods:**

Cross-sectional study was conducted in South west Ethiopia from January 15 – March 20, 2012. A sample of youths aged 15–24 years was taken from six health centers and three hospitals using systematic sampling technique. Cox proportional hazard regression model was used to assess the association between the outcome and explanatory variables.

**Result:**

A total of 405 youths participated in the study and the mean ( ±SD ) age of sexual initiation was 17.07 years (±2.12). Age at first sexual initiation was positively associated with lack of employment [Adj. HR & (95% CI) = 7.372 (1.455, 37.357)], lack of comprehensive knowledge on HIV [Adj. HR & (95% CI) = 8.247 (2.121, 32.067)], alcohol use [Adj. HR & (95% CI) = 3.815 (1.315, 11.070)] and khat use [Adj. HR & (95% CI) = 7.241 (1.871, 28.016)].

**Conclusion:**

Majority of the study participants experienced sexual initiation. Strategies should be designed to control the use of substances like alcohol and khat which were found to be responsible for first sexual initiation.

## Background

The transition to adolescence is a period of rapid change and young adolescents seek peer connections to obtain a stable point of reference during this time [1]. Young people in rural areas are also increasingly migrating to urban centers and migration increases the risks of exploitation and sexual violence [2]. Youths are particularly vulnerable to HIV in Ethiopia with a prevalence of 2.4% as evidenced from the 2005 demographic and health survey report and are the key to the future course of the HIV/AIDS epidemic. Since most new infections are in young people, they have been given higher priority in the prevention and control of HIV/AIDS in Ethiopia [3]. Delaying sexual debut is the pillar of HIV/STIs prevention among young people.

Early sexual debut increases young peoples’ risk for infection with HIV and other STIs. Youth who begin early sexual activity are more likely to have high-risk sex or multiple partners and are less likely to use condoms [4]. According to Ethiopian Demographic and Health Survey (EDHS) 2005; among women age 25–49 years, 32% had sexual intercourse before age 15 years, 65% before age 18 years, and by age 25 years most Ethiopian women have had sexual intercourse. In Ethiopia, trends in the median age at first sexual intercourse has increased little over the past five years from 16.5 years in 2005 to 17.1 years in 2011 for women and from 21.1 years in 2005 to 21.2 years in 2011 for men [5,6]. The median age of marriage for women in Ethiopia is 17.1 years indicating that for most girls, marriage drives sexual debut [5].

As evidenced by different literatures timing of sexual debut among youths is influenced by a wide range of factors including age, gender, residence, educational level, knowledge on HIV, economic status, watching pornography, Khat and alcohol utilization [7-16]. Young girls in Ethiopia are more vulnerable to HIV than boys because of early age at sexual debut, early marriage, sexual abuse and violence such as rape and abduction [6]. Early childbearing has been linked to higher rates of maternal and child morbidity and mortality, truncated educational opportunities, and lower future family income, larger family sizes, which in turn may lead to greater population growth [5,11]. Higher rates of unintended pregnancy, abortion, HIV/AIDS and other sexually transmitted infections among youths make it crucial for a need to understand and assess the factors that are associated with early sexual initiation. Therefore, the main aim of this study was to determine the time of first sexual initiation and associated factors among youths in south west Ethiopia.

## Methods

### Study setting and period

Health institution based cross-sectional study was conducted from January 15 – March 20, 2012. The study was conducted in Gamo Gofa zone which is located about 505 km south west of Addis Ababa the capital of Ethiopia. Gamo Gofa is located in the Ethiopian Southern Nations, Nationalities and Peoples Region (SNNPR). It is named for the Gamo and Gofa peoples whose homelands lie in this area and the administrative center of Gamo Gofa is Arba Minch. According to the 2007 census result it has a population of 1,595,570 and of this 794,485 were males and 801,085 were females. There are a total of three hospitals and 68 health centers out of which all three hospitals and twenty four health centers offer HIV testing and counseling service for the total population. The study population was all youths of 15–24 years attending HIV testing and counseling centers in public health facilities during the data collection period.

### Sampling procedure

Three hospitals and three health centers offering HIV testing and counseling service in Gamo-Gofa zone was included in the study. Health institutions were stratified into health center and hospital and in each stratum systematic sampling technique was used to select the respondents. Based on the number of clients who visited each health institution during the previous one year (annual report of each health institution), the total sample size was proportionally divided to each stratum (41.5% for health centers and 58.5% for hospitals). Youths in the age range of 15–24 years were included in the study and those who are unable to communicate (having hearing problem and cannot communicate with sign language) were excluded from the study.

### Data collection and quality control

Data were collected using structured questionnaires during the pre-test counseling period. Any error and incompleteness were corrected before the individual was told the test result in his/her visit for the post-test counseling. Six data collectors and three supervisors who were HIV counselors from the respective health institutions were recruited. Two days training was given for data collectors and supervisors by investigators on how to fill out the questionnaire during the pre-test counseling and correction of any ambiguity or incompleteness during the post-test counseling. Trustworthiness of the data was assured by giving training for data collectors and by using immediate and daily supervision. The collected data was checked for completeness and consistency by supervisors and principal investigators every day. The questionnaire was first prepared in English and it was translated to the local language (Amharic) and back to English in order to maintain the validity of the instrument. The questionnaire was pretested two weeks before the actual data collection to ensure its clarity and understandability. After final modification, the questionnaire was used to elicit the following information from the study participants: socio-demographic variables, comprehensive knowledge on HIV, alcohol use, khat use, peer pressure, number of sexual partners, use of condom and viewing pornographic materials which were independent variables in this study. Data coding, cleaning and verification was performed before the data was entered in to SPSS version 16.

### Operational definitions

#### Comprehensive knowledge on HIV

Was measured by ability to identify the two important prevention ways (being faithful and condom use), being aware that a healthy-looking person can have HIV and reject the two locally common misconceptions about HIV transmission (mosquito bite and sharing food).

#### Alcohol use

A study participant was considered as ever drinker if he/she responds yes to the question ‘Have you ever drunk alcohol in your life?’ Then follow up questions were employed to collect information such as drinking in the past one year and frequency of drinking. Current alcohol use is defined as use of alcohol at least once during the past 30 days before the survey.

#### Khat use

Participants were considered as ever chewer if he/she responds yes to the question ‘Have you ever chewed khat in your life?’ Then follow up questions were employed to collect information such as khat chewing in the past one year and frequency of chewing. Current khat use is defined as use of khat at least once during the past 30 days before the survey.

#### Early sexual initiation

Was defined as having sexual intercourse before the age of 18 years and all these operational definitions were adopted from the Ethiopian demographic and health survey 2011 [6].

#### Planned or non-casual sex

Was defined as certain types of human sexual activity in the context of a romantic relationship. It excludes extramarital sex or sex in a casual relationship.

#### Employment

Was measured by asking the study participants if they are recruited as an employee in any organization for any type of work especially for those who are out of school either because of school dropout or completion of their education.

#### Monthly income

Was measured by asking the monthly income of employed participants and for those who are students the monthly income of their family was asked and used as a proxy measure.

### Data processing and analysis

Sample size was determined by using the formula of single population proportion estimation by taking 41% proportion [5], 5% of absolute precision and with 95% confidence interval. Non-response rate in this study was estimated to be 10% and hence an overall sample size of 410 youths were recruited in the study. Data were entered and analyzed using SPSS software version 16. Descriptive statistics such as frequencies and proportion was used to describe the study population in relation to relevant variables. Kaplan-Meier curves or log-rank test were used to select variables to enter into the multivariate Cox proportional hazard regression model. Variables having a p-value of ≤ 0.2 in bi-variate analysis were entered into the multivariate Cox regression analysis by taking not having sex as an outcome variable and age as a follow up period. Crude and adjusted hazard ratio (HR) with p-values and 95% CI were computed to measure the association of age at first sexual initiation with explanatory variables.

### Ethical consideration

Ethical clearance was obtained from ethical review board of Arba-Minch University and permission to conduct the study in each health facilities was secured from the respective health institutions in Gamo-Gofa. Verbal informed consent from each study participant was obtained after clear explanation about the purpose of the study. Written informed consent sheet was prepared for the parents or legal guardians of participants less than 18 years to give their signed consent on behalf of their children. The study participants and parents or legal guardians were informed that they have full right to withdraw from the study without losing any of their right as a client in the health institutions. Confidentiality of the information was assured by omitting names of study participants from the questionnaire and respondents were interviewed in a separate room to maintain their privacy.

## Results

A total of 405 youths participated in this study. About 301 (74.3%) of the respondents were from urban areas. Two hundred seventy six (68.1%) of the respondents were never married and 299 (73.8%) of them had secondary and above education level. Most 183 (45.3%) of them were Gamo by ethnicity and 221 (54.6%) were followers of orthodox Christianity followed by protestant 158 (39.0%). Majority of the study participants 294 (72.6%) were in the age range of 20–24 years with mean (±SD) age of 20.57 (± 2.59) Table 1.

**Table 1:** Socio-demographic characteristics of youths visiting HIV testing and counseling centers in Gamo Gofa, South West Ethiopia, 2012

**Variables**	**Number**	**Percent**
**Age** [Mean(±SD) = 20.57(±2.59)]		
15 - 19	111	27.4
20 - 24	294	72.6
**Sex**		
Male	186	45.9
Female	219	54.1
**Residence**		
Urban	301	74.3
Rural	104	25.7
**Ethnic group**		
Gamo	183	45.3
Gofa	114	28.2
Amhara	41	10.1
Others*	66	16.3
**Religion**		
Orthodox Christian	221	54.6
Muslim	18	4.4
Protestant	158	39.0
Others**	8	2.0
**Education**		
No education	59	14.6
Primary Education	47	11.6
Secondary & above	299	73.8
**Occupation**		
Government employed	73	18.0
Merchant	50	12.3
House wife	31	7.7
Daily Laborer	54	13.3
Student	19	48.6
**Marital Status**		
Never Married	276	68.1
Married/living together	99	24.4
Divorced/separated/ widowed	30	7.4
**Monthly income**		
<= 450 Ethiopian birr ( <= $ 25 )	243	60.0
451 – 999 Ethiopian birr ( $ 25.1 - $ 55.5 )	72	17.8
> = 1000 Ethiopian birr ( > = $ 55.6 )	90	22.2

* Wolayta, Konso, Gurage, Oromo ** Catholic, Adventist, Only Jesus.

### Mean age of sexual initiation and non-sexual behaviors of the study participants

Seventy two percent of the study participants had initiated sex out of which 52.7% of first sex was casual. The mean (±SD) age of sexual initiation in this study was 17.07 years (2.12). About forty four percent of youths have heard of pornography and fifteen percent of them had been exposed to pornography. The prevalence of alcohol and khat use was found to be 43.5% and 27.4% respectively (Table 2).

**Table 2:** Sexual and non-sexual behaviors of youths visiting HIV testing and counseling centers in Gamo Gofa, South West, Ethiopia, 2012

**Variables**	**Number**	**Percent**
**Ever had sex**		
Yes	292	72.1
No	113	27.9
**Age at first sex** [Mean(±SD) = 17.07(±2.12)]		
< 18 years	166	56.9
> = 18 years	126	43.1
**First sex was:**		
Non-casual	138	47.3
Casual	154	52.7
**Know pornography**		
Yes	180	44.4
No	225	55.6
**Exposed to pornography**		
Yes	63	15.6
No	342	84.4
**Alcohol use**		
Yes	176	43.5
No	229	56.5
**Khat use**		
Yes	111	27.4
No	294	75.6

### Predictors of Age at first sexual initiation among youths in gamo gofa, south west Ethiopia

Variables entered into the multivariate Cox regression analysis were; Age, sex, employment, educational status, comprehensive knowledge on HIV, Khat use, alcohol use, exposure to pornography and HIV status. Cox regression analysis showed that unemployed youths were 7.4 times [Adj. HR & (95% CI) = 7.4 (1.5, 37.4)] as likely to initiate sex. Youths who do not have comprehensive knowledge on HIV were 8.3 times [Adj. HR & (95% CI) = 8.3 (2.1, 32.1)] more likely to initiate sex earlier than those who do have. Compared to youths who do not use alcohol, those who are alcohol users were 3.8 times [Adj. HR & (95% CI) = 3.8 (1.3, 11.1)] as likely to initiate sex earlier. In like manner, Khat users were 7.2 times [Adj. HR & (95% CI) = 7.2 (1.871, 28.016)] more likely to initiate sex earlier than their counterparts Table 3.

**Table 3:** Cox regression indicating factors associated with age at first sexual initiation among youths (Crude & adjusted hazard ration) in Gamo Gofa, South West Ethiopia, 2012

**Explanatory variables**	**Crude HR (95% CI)**	**Adjusted HR (95% CI)**	**P-value**
**Employed**			
Yes	1.00	1.00	0.02
No	1.2 (0.85, 1.6)	7.4 (1.5, 37.4)	
**Have comprehensive knowledge on HIV**			
Yes	1.00	1.00	0.01
No	1.3 (0.91, 1.4)	8.3 (2.1, 32.1)	
**Alcohol use**			
Yes	1.3 (0.97, 1.8)	3.8 (1.3, 11.1)	0.01
No	1.00	1.00	
**Khat use**			
Yes	1.1 (0.76, 1.5)	7.2 (1.9, 28.0)	0.01
No	1.00	1.00	
**Exposed to pornography**			
Yes	1.2 (0.95, 1.5)	1.9 (1.0, 3.3)	0.09
No	1.00	1.00	

## Discussion

The mean age of first sexual initiation (17.07) in this study is slightly higher than previous studies done in Dessie, north east Ethiopia (16.8 years), in Kolladiba, north west Ethiopia (15 years), in Gojam, north west Ethiopia (13.5 years) and in Butajira, southern Ethiopia (16 years) [11,17-19]. The difference may be explained by the decrease in early marriage which was the main reason for early sexual initiation in rural youths [5,6,11,17]. Another explanation might be AIDS-related campaigns to delay first sexual intercourse might have had an inhibiting effect [5,6,16].

Early entry to sexual initiation has very important implications for the sexual and reproductive health of youths. In this study alcohol users were almost four times more at risk to initiate sexual intercourse earlier than those who didn’t use alcohol and this is in line with studies done in other parts of Ethiopia [15,17]. This is true even despite the concern about HIV infection, because alcohol decreases self control and sexual negotiation skill of adolescents [11,13]. Youths who chewed khat were also found to be seven times more likely to initiate sexual intercourse earlier than their counterparts. This finding is also supported by similar studies in other parts of Ethiopia [11,15,17]. The possible explanation for this association could be due to loss of track of mind induced by khat chewing. During the hypo manic phase, chewers may not be capable of rational judgment and they also may not be able to predict the serious consequences of their actions [13,15]. Thus, the chewers could walk into the most dangerous situations feeling that there is no danger and being unaware of the possible dangers to their lives or well-being, they get motivated to have casual and early sexual initiation [13-15].

This study also indicated that unemployed youths were almost seven times more at risk to practice early sexual initiation than those who are employed. The higher risk of early sexual initiation among unemployed youths in our study raises our suspicion of the use of alcohol and khat by unemployed youths which predisposed them to have early sexual initiation. This possible explanation is supported by a study in Ethiopia in which the most frequent substance abusers were jobless youths and street children [12].

Youths with no comprehensive knowledge on HIV were found to be eight times more likely to practice early sexual initiation than those who have comprehensive knowledge on HIV and this could be due to the fact that youths who do not have comprehensive knowledge on HIV may fail to appreciate the importance of delay of sexual intercourse for prevention HIV infection or may have less access to sexual and reproductive health education and promotion in general. This is in line with the research finding from Nigeria where youths with poor knowledge on HIV initiated sex earlier compared to youths with good knowledge on HIV [16] and another study among Flemish secondary school students reported similar finding [20].

However viewing pornographic materials were not associated with early sexual initiation in this study which contrasts with the result of a study among college students in Korea where youths having pornography experience were at higher risk of initiating sex than their counterparts [14]. This might be due to few numbers of respondents who acknowledged exposure to pornographic materials in our study populations. The possible explanation for this could be under-reporting of exposure to pornographic materials because of cultural issues or could be due to recall bias.

Social desirability bias is a potential limitation of this study. Another limitation is the use of cross sectional data and this makes it difficult to establish causality. The study is also health facility based and therefore precludes generalization to all youths in Ethiopia. Despite these limitations, the study provides useful information that will inform health service planners to design a strategy for delaying age at first sex and for the prevention of HIV in Ethiopia.

## Conclusion

Majority of the study participants experienced sexual initiation. Independent predictors of early sexual initiation were luck of employment, poor comprehensive knowledge on HIV, alcohol and khat use. Strategies should be designed to control the use of substances like alcohol and khat which were found to be responsible for early sexual initiation in this study. Youth friendly centers are recommended for those individuals who are sexually active.

## Competing interests

The authors declare that they have no competing interests.

## Authors’ contributions

MT was investigator, involved in proposal writing, designing, and recruitment and training of supervisors and data collectors, analysis and write-up and in all stages of the project implementation. He did most of the analysis and write up of the paper. GA contributed in the designing of the methodology, recruitment and training of supervisors and data collectors and involved in designing of project proposal, design of questionnaires, supervision and involved in the final approval of the paper. All authors read and approved the final manuscript.

## Pre-publication history

The pre-publication history for this paper can be accessed here:

http://www.biomedcentral.com/1471-2458/13/622/prepub
